# Synthetic OCT Data Generation to Enhance the Performance of Diagnostic Models for Neurodegenerative Diseases

**DOI:** 10.1167/tvst.11.10.10

**Published:** 2022-10-06

**Authors:** Hajar Danesh, David H. Steel, Jeffry Hogg, Fereshteh Ashtari, Will Innes, Jaume Bacardit, Anya Hurlbert, Jenny C. A. Read, Rahele Kafieh

**Affiliations:** 1 School of Advanced Technologies in Medicine, Medical Image and Signal Processing Research Center, Isfahan University of Medical Sciences, Isfahan, Isfahan, Iran; 2 Sunderland Eye Infirmary, Sunderland, Tyne and Wear, UK; 3 Centre for Transformative Neuroscience and Institute of Biosciences, Newcastle University, Newcastle upon Tyne, Tyne and Wear, UK; 4 Royal Victoria Infirmary Eye Department, Newcastle Upon Tyne Hospitals NHS Foundation Trust, Newcastle Upon Tyne, Newcastle Upon Tyne, UK; 5 Population Health Sciences Institute, Newcastle University, Newcastle Upon Tyne, Tyne and Wear, UK; 6 Isfahan Neurosciences Research Center, Isfahan University of Medical Sciences, Isfahan, Iran; 7 Translational and Clinical Research Institute, Newcastle University, Newcastle Upon Tyne, Tyne and Wear, UK; 8 School of Computing, Newcastle University, Newcastle upon Tyne, Tyne and Wear, UK; 9 Department of Engineering, Durham University, South Road, Durham, UK

**Keywords:** synthesis model, augmentation, optical coherence tomography, model validation

## Abstract

**Purpose:**

Optical coherence tomography (OCT) has recently emerged as a source for powerful biomarkers in neurodegenerative diseases such as multiple sclerosis (MS) and neuromyelitis optica (NMO). The application of machine learning techniques to the analysis of OCT data has enabled automatic extraction of information with potential to aid the timely diagnosis of neurodegenerative diseases. These algorithms require large amounts of labeled data, but few such OCT data sets are available now.

**Methods:**

To address this challenge, here we propose a synthetic data generation method yielding a tailored augmentation of three-dimensional (3D) OCT data and preserving differences between control and disease data. A 3D active shape model is used to produce synthetic retinal layer boundaries, simulating data from healthy controls (HCs) as well as from patients with MS or NMO.

**Results:**

To evaluate the generated data, retinal thickness maps are extracted and evaluated under a broad range of quality metrics. The results show that the proposed model can generate realistic-appearing synthetic maps. Quantitatively, the image histograms of the synthetic thickness maps agree with the real thickness maps, and the cross-correlations between synthetic and real maps are also high. Finally, we use the generated data as an augmentation technique to train stronger diagnostic models than those using only the real data.

**Conclusions:**

This approach provides valuable data augmentation, which can help overcome key bottlenecks of limited data.

**Translational Relevance:**

By addressing the challenge posed by limited data, the proposed method helps apply machine learning methods to diagnose neurodegenerative diseases from retinal imaging.

## Introduction

Multiple sclerosis (MS) is an unpredictable and recurrent disease that affects the nerve cells of the brain and spinal cord and destroys the protective myelin sheath around the nerve fibers, causing vision problems and impaired muscle control. Loss of vision is one of the leading causes of disability in patients with MS. Several studies have reported a correlation between the axonal loss in the optic nerve and the degree of functional disability in patients with MS.^1^^,^^2^ Neuromyelitis optica (NMO) is another neurodegenerative disease that affects the eye and spinal cord and occurs when the immune system attacks healthy cells in the central nervous system.

Optical coherence tomography (OCT) facilitates cross-sectional imaging of the retina based on interference patterns produced by low-coherence near-infrared light reflected from retinal tissues. OCT has been used as an easy, fast, and noninvasive method for qualitative and quantitative evaluation of retinal changes in neurologic disorders.^3^^,^^4^ This technique makes it possible to reconstruct cross-sectional structural images with an axial resolution of approximately 4 µm.^5^

Over the past decades, several clinical and paraclinical procedures have been performed to diagnose neurodegenerative diseases like MS and NMO. Magnetic resonance imaging (MRI) is widely used to diagnose specific inflammatory lesions and tissue atrophy in the brain and spinal cord.^6^^,^^7^ Recently, diagnostic procedures have been increasingly complemented by retinal imaging with OCT, first described in MS by Parisi et al.^8^ Further studies have shown that two features derived from OCT scans in MS and NMO—namely, the peripapillary retinal nerve fiber layer thickness as a measure of axonal health and the macular volume and ganglion cell and inner plexiform layer (GCILP) thickness as a measure of neuronal health—are linked to MRI-based measures of myelin health in the posterior visual pathway.^9^^–^^16^

Machine learning (ML) methods have great promise in ophthalmology for discriminating different diseases.^17^^,^^18^ The main limitation of ML in applications like discrimination of MS and NMO is the availability of large and well-annotated training data sets. Synthetic OCT data could address this issue by supplying additional training data, covering underrepresented classes to reduce bias, and avoiding the privacy issues associated with the collection of real imaging data.

**Figure 1.**
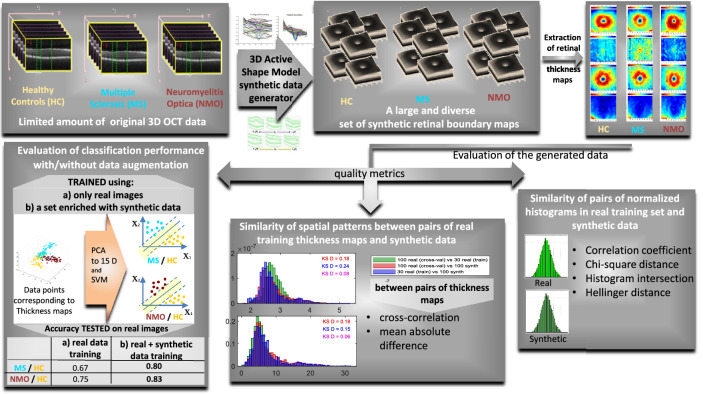
Graphical abstract of the proposed approach.

The construction of synthetic OCT data has been considered by researchers over the past few years.^19^^–^^22^ In recent work,^23^^,^^24^ we used an active shape model (ASM^25^) to construct synthetic two-dimensional (2D) and three-dimensional (3D) OCT data in the macular region. In this article, we use that model as an augmentation method to generate synthetic 3D OCT boundaries of the macular region from healthy controls (HCs) and patients with MS and NMO. The thickness maps of retinal layers (strong biomarkers of MS and NMO) are then calculated using both synthetic and real data. Three validation strategies are formulated to assess the utility and integrity of the generated data and to justify its use to augment real data in future research. The strategies include histogram comparison methods, comparison of statistical properties between original 2D maps (retinal thickness maps), and a standard classification measure to evaluate the efficacy of the synthetic data augmentation method in disease prediction. Figure 1 shows the proposed approach in a graphical abstract.

## Materials and Methods

The OCT data set was collected using Spectralis SD-OCT (Heidelberg Engineering, Heidelberg, Germany) at the Kashani Comprehensive MS center, Isfahan, Iran (details in Ashtari et al.^14^). It consists of OCT data from HCs (26 eyes) and patients with NMO (30 eyes) and MS (30 eyes). To construct the proposed model, a limited number (five OCT volumes) were randomly selected from each class to be used in the training stage and to synthesize 25 three-dimensional OCT boundaries in each category. In total, 130 OCT volumes from HCs^26^ were used for further validation of the synthetic data (30 eyes for training and 100 eyes for further comparison with synthetic data).

Each OCT image stack contains 25 horizontal B-scans (each with 512 A-scans, with automatic real-time tracking in nine frames and axial resolution of 3.8 mm), scanning a macular area of 6 by 6 mm focused on the fovea. Automated segmentation of retinal layer boundaries was performed using a custom-developed graph-based method^27^ with reference values presented in Kafieh et al.^26^ The segmentation results were quality controlled and manually corrected in case of errors by an ophthalmologist using the method in Montazerin et al.^28^ To account for eye laterality, 3D OCTs from left eyes are flipped and the nasal area is located on the right side of the thickness maps.

**Figure 2.**
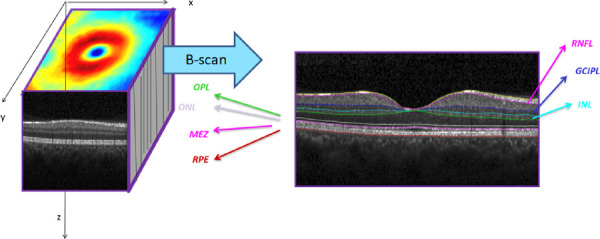
Example of retinal 3D OCT image stack and the location of retinal layer boundaries.

The boundaries of intramacular layers were calculated for macular retinal nerve fiber layer (mRNFL), GCIPL, inner nuclear layer (INL), outer plexiform layer (OPL), outer nuclear layer (ONL), myoid-ellipsoid zone (MEZ), and retinal pigment epithelium (RPE). The reporting is according to APOSTEL recommendations.^29^ Figure 2 shows an example of a 3D OCT image stack with extracted layers.

### Synthetic Data Generation Using 3D ASM

Generating synthetic data requires a statistical shape model that can produce new 3D retinal layer boundaries. The model should be general (i.e., able to generate any plausible example of the class it represents), so the thickness and morphology of the synthetic retinal layers must be sufficiently variable. However, the model should also be target oriented, which means that it is only authorized to generate suitable shapes. To achieve this, a statistical model based on prior knowledge is built by analyzing the statistical characteristics of a set of annotated 3D OCT scans in the training stage. The annotations provide a set of feature points in three dimensions by which the training maps are aligned and from which the model extracts their principal components. The trained model captures and generalizes the statistical characteristics of the retinal layer boundaries in the training set, allowing us to synthesize similar layer boundary shapes.

Annotated 3D OCT scans in the training stage are first cropped to cover a symmetrical 6-mm distance around the fovea. The layer boundaries in the training set must cover the different types of variation we wish the model to represent. We annotate each 3D OCT image stack with a set of points used as landmarks for the alignment. In this study, we use 91 landmark points on each of the eight retinal layer boundaries on each of the 25 B-scans in a stack, for a total of *n* = 18,200 points per image stack.

For the *i*th boundary, the *j*th landmark point is represented by (*x_ij_*, *y_ij_*, *z_ij_*), in coordinates where *x,y* corresponds to the horizontal and vertical components of each B-scan, *z* indexes the identity of each B-scan, and *j* runs from 1 to 91. By definition, *z_ij_* is the same for all points in a given B-scan. The first and last landmark points are taken to be the left and right edges of the B-scan; thus, by definition, *x_i_*_1_ = 1 and *x_i_*_91_ = 512 in every case, with the values *y_1_*_1_ and *y_i_*_91_ giving the height of the *i*th boundary at the edges. To obtain the other landmark points, we identify the coordinates corresponding to the center of the macula in the given image stack (*x*_mac_*,y*_mac_*,z*_mac_). For all boundaries, *x_i_*_46_ is defined to be *x*_mac_. The remaining points *x_i_*_2_… *x_i_*_45_ and *x_i_*_47_… *x_i_*_90_ are then spaced evenly between, respectively, *x_i_*_1_ = 1 and *x_i_*_46_ = *x*_mac_, and *x_i_*_46_ = *x*_mac_ and *x_i_*_91_ = 512. Each *y_ij_* is then the height of the *i*th boundary at *x_ij_*.

We apply Procrustes analysis^30^ to align all image stacks to a reference image stack, and a point distribution model^31^ is then constructed. Assuming that the variability within the population occurs along just a few directions in this space, the dimensionality is reduced to a lower space using principal component analysis (PCA). Each layer boundary in the training set can now be approximated by the mean shape plus a weighted sum of the first *t* principal components, and we can synthesize new layer boundaries by allocating different numbers to weights of plausible principal components. Details of this procedure are elaborated in the Supplementary Material.

In this way, we generate synthetic vectors ***X***, describing the locations of the 18,200 landmark points on layer boundaries of a synthetic OCT stack. We finally interpolate values linearly between the landmark points to recover the complete synthesized boundaries.

**Figure 3.**
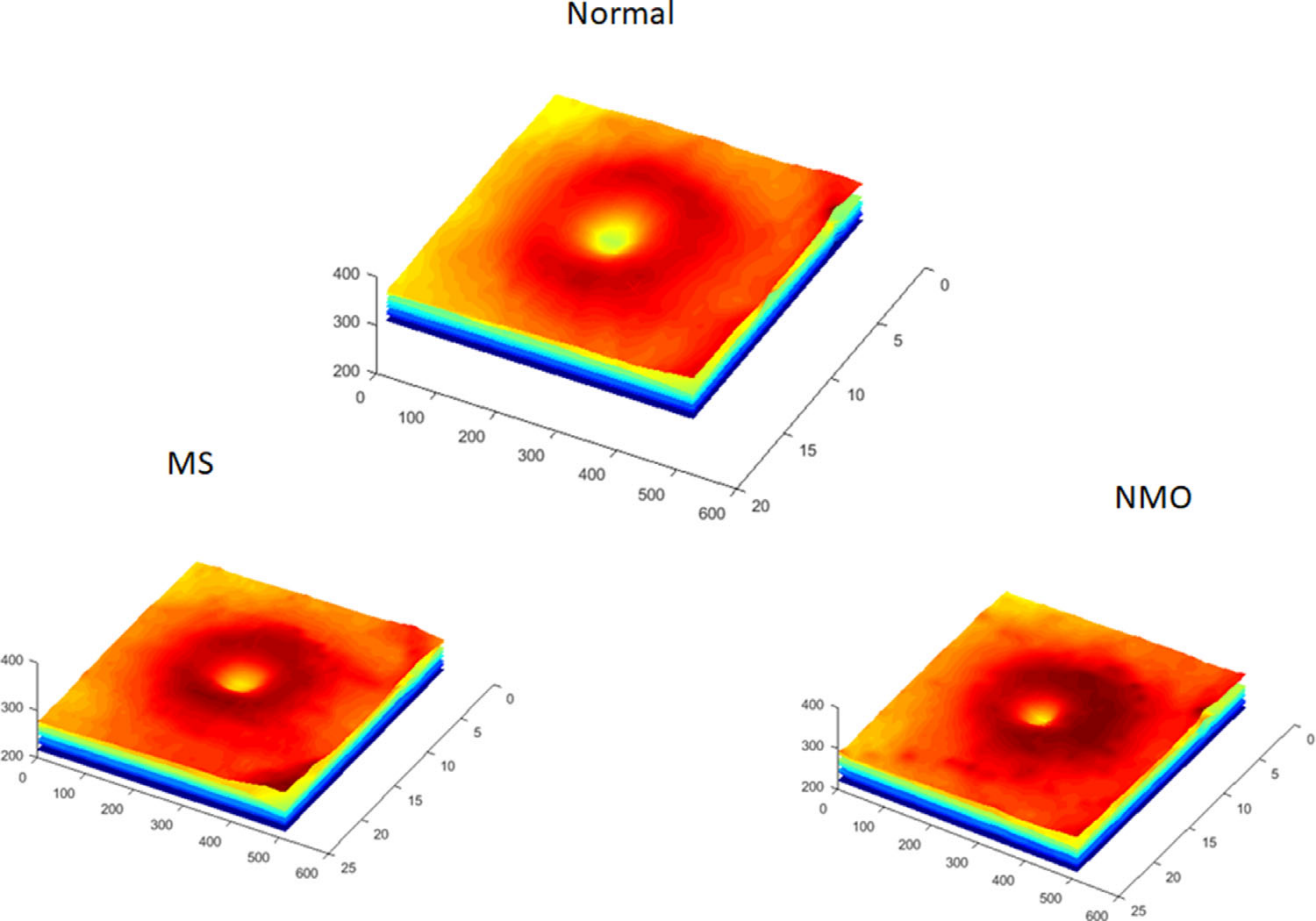
Example of synthetic retinal layer boundaries in each class (HC, MS, and NMO). The color codes the height (y-value) of each boundary point.

Figure 3 shows examples of the synthesized layer boundaries for each of the classes (HC, MS, and NMO). As we focus on retinal thickness maps because of their usefulness as biomarkers for neurologic diseases, it is not necessary to create synthetic textures in order to generate a full OCT image, as we did in Danesh et al.^24^

### Construction of Retinal Thickness Maps

**Figure 4.**
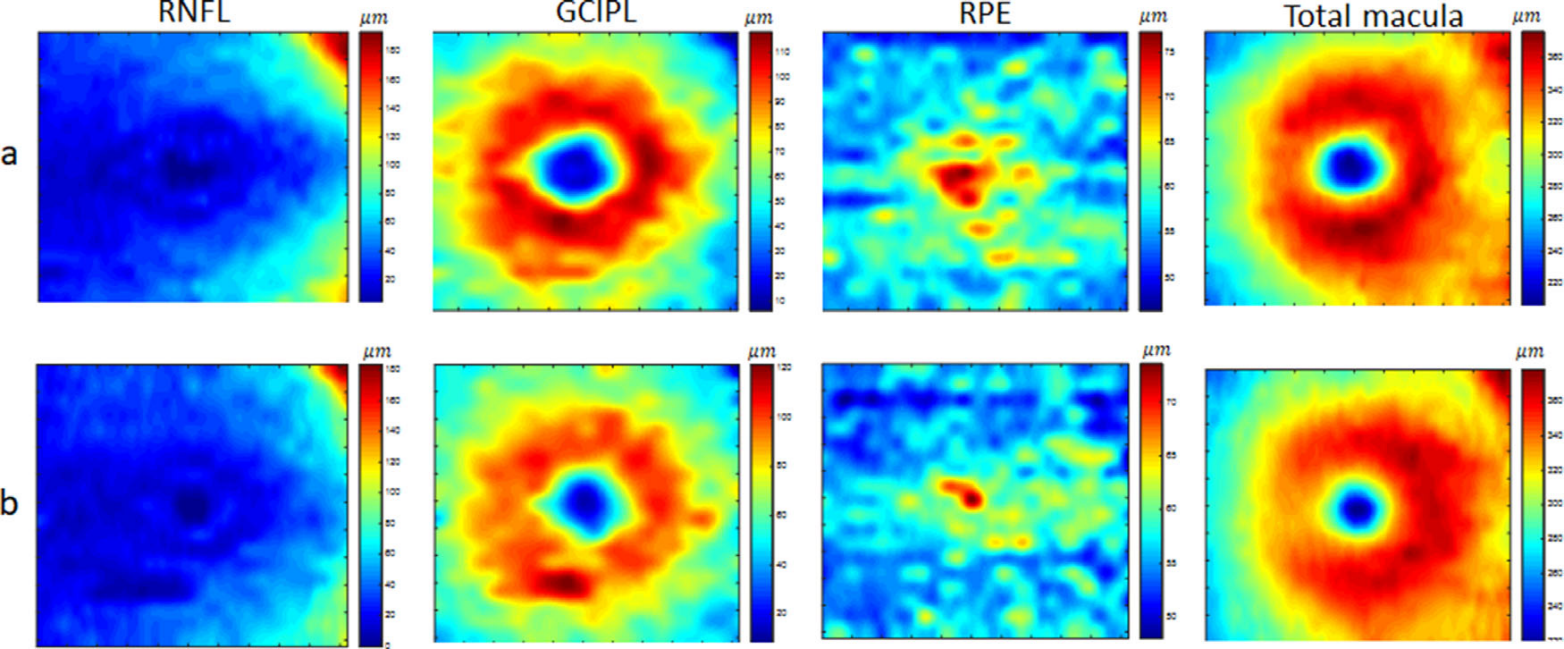
Example of thickness maps from mRNFL, GCIPL, RPE, and the total retina in an area of 6 by 6 mm around the fovea in the HC class. The *upper row* (a) shows the real data, and the *lower row* (b) shows the synthetic data with the proposed method.

**Figure 5.**
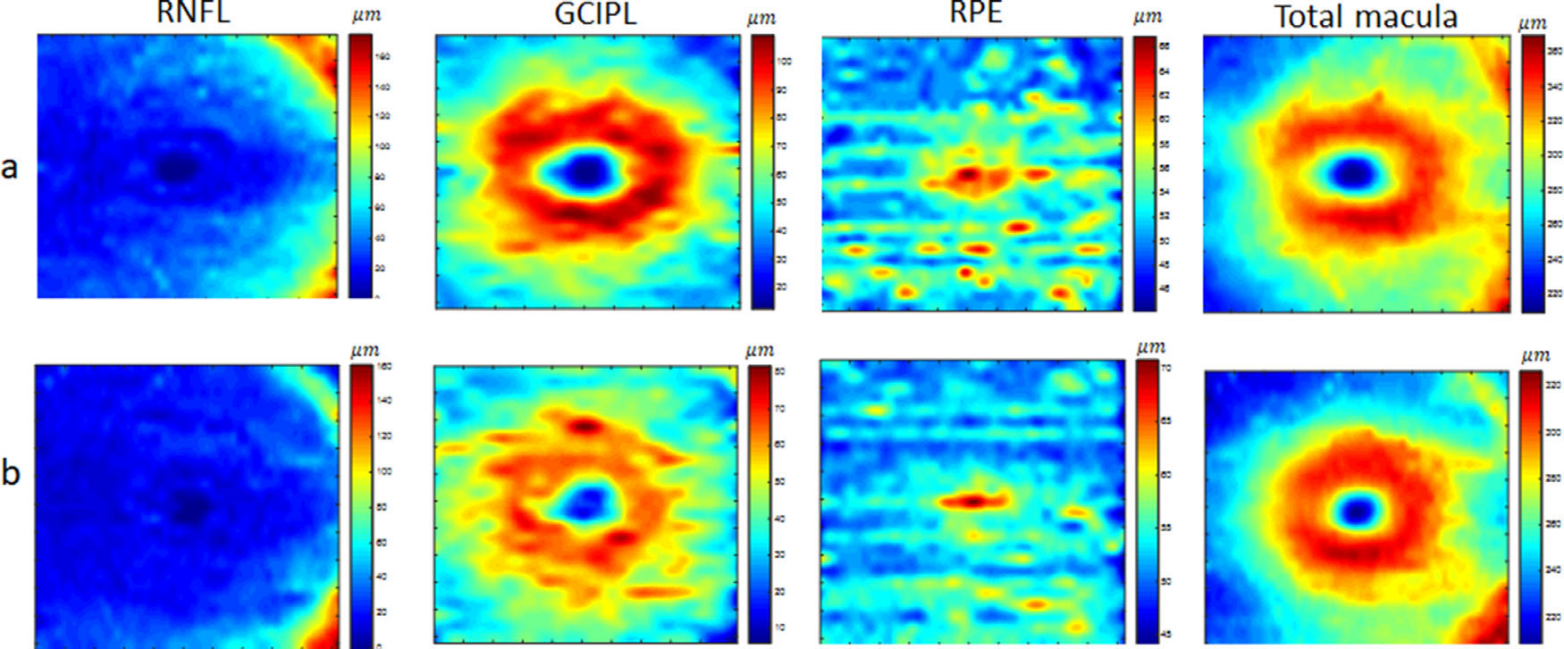
Example of thickness maps from mRNFL, GCIPL, RPE, and the total retina in an area of 6 by 6 mm around the fovea in the MS class. The *upper row* (a) shows the real data, and the *lower row* (b) shows the synthetic data with the proposed method.

**Figure 6.**
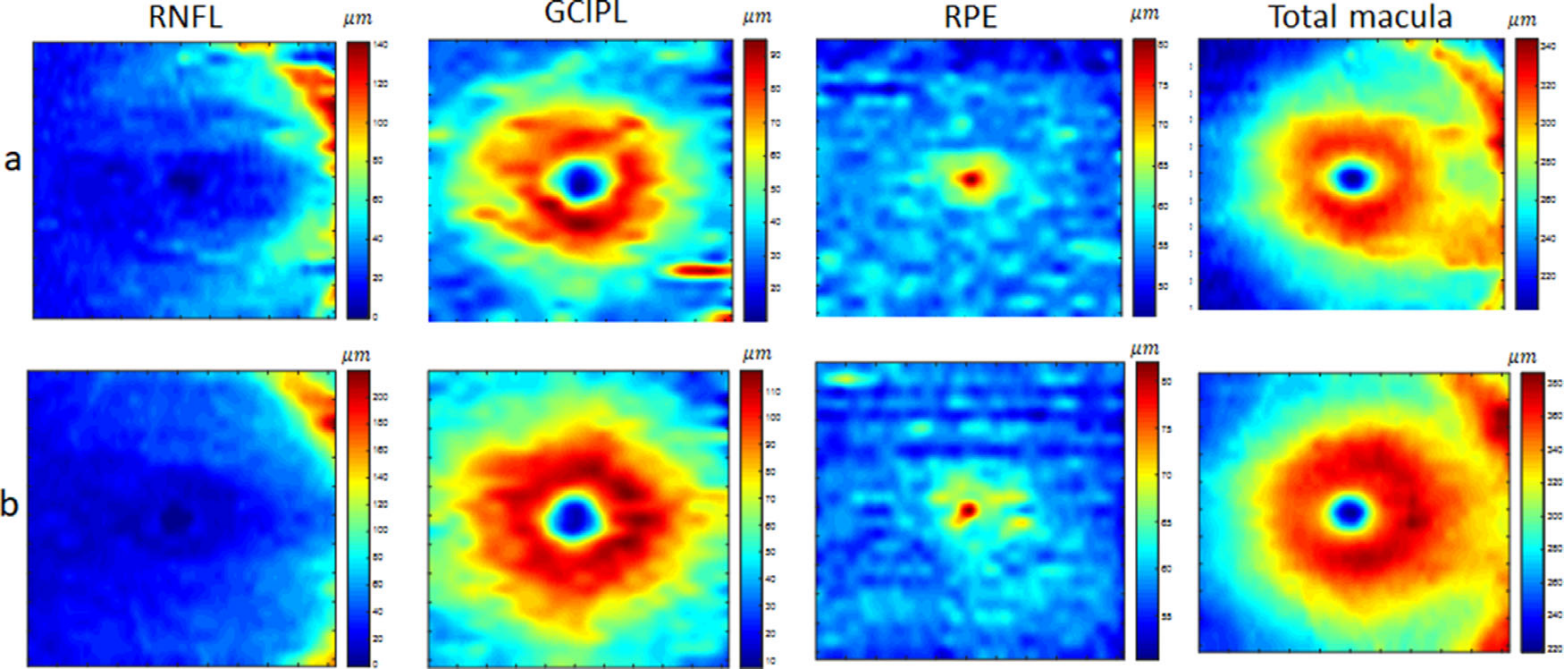
Example of thickness maps from mRNFL, GCIPL, RPE, and the total retina in an area of 6 by 6 mm around the fovea in the NMO class. The *upper row* (a) shows the real data, and the *lower row* (b) shows the synthetic data with the proposed method.

Retinal thickness maps have a potential role in the diagnosis of neurodegenerative diseases.^32^^–^^34^ They reveal information implicit in the 3D OCT volumes by providing easily interpretable maps for each retinal layer. We, therefore, calculated the thickness of each retinal layer and of the whole retina as 2D maps to be used in the validation strategies discussed below. The thickness of each retinal layer is the distance between consecutive retinal boundaries; similarly, the thickness of the entire retina is obtained as the distance between the first and the last boundaries. Accordingly, macular thickness maps are calculated for all three data classes, demonstrated in Figures 4, 5, and 6 for selected retinal layers (mRNFL, GCIPL, RPE) and the total retinal thickness. The real thickness maps and corresponding synthetic maps are shown in the first and second rows, respectively. Each thickness map has a size of 512 × 25 pixels, but the examples in Figures 4 to 6 are resized to 500 × 500 pixels for better visualization.

### Validation Strategies

Three validation strategies are employed to check the usefulness and integrity of the synthesized data and to justify its use as an augmentation method in future research. The first two are statistical. First, we compute image histograms of the thickness maps for a given layer and compare these in real and synthesized maps. This checks that the range of thicknesses is comparable but does not evaluate their spatial pattern. To achieve that, we examine the cross-correlation and mean absolute difference between pairs of thickness maps and compare the distribution obtained for pairs of real maps with that obtained when one image is real and the other synthetic. The last strategy builds classification models to discriminate between HCs, MS, and NMO in order to assess our synthetic data generator as a data augmentation method. We compare the predictive performance of models trained using only real maps versus models trained from a set enriched with our synthetic data.

#### Histogram-Based Validation

This validation strategy checks the correspondence between the histogram of the generated 2D maps (retinal thickness maps) and the histogram of the maps in the training set. For this purpose, retinal thickness maps are calculated for retinal layers (mRNFL, GCIPL, INL, OPL, ONL, MEZ, and RPE). For each retinal layer with a thickness map of 512 × 25 pixels, the histogram is calculated to represent the distribution of thickness values by counting how many pixels out of 512 × 25 fall into each thickness interval. The histogram is then normalized to display relative frequencies as a proportion of pixels that fall into each of several thickness categories, with the sum of the heights equaling 1. The normalized histogram can then be interpreted as discretized probability distributions whose value at any given thickness provides a relative likelihood that the value of the random variable (pixels of the 2D image of thickness map) would be close to that sample thickness. To quantify the similarity of pairs of normalized histograms in the real training set (*H*_1_) and synthetic data (*H*_2_), four measurements are used.

i. The correlation coefficient is used to determine the type (direct or inverse) and degree of the relationship between two discretized probability distributions, approximating the similarity of the histograms. This coefficient ranges between 1 and –1 (zero if no correlation exists):
  (1) $$\begin{array}{ccc} & & C\left(H_{1},H_{2}\right)\\ & & =\frac{\sum_{I}\left(H_{1}\left(I\right)-\bar{H_{1}}\;\right)\left(H_{2}\left(I\right)-\bar{H_{2}}\;\right)}{\sqrt{\sum_{I}{\left(H_{1}\left(I\right)-\bar{H_{1}}\;\right)}^{2}\sum_{I}{\left(H_{2}\left(I\right)-\bar{H_{2}}\;\right)}^{2}}} \end{array}$$
  where $\bar{H_{i}},\;i=\left[1,2\right]$ is the mean value of each histogram over the total number of histogram bins, and *I* denotes the bin number.
ii. The chi-square distance calculates the normalized square difference between two histograms, and for identical histograms, this distance equals zero:
  (2) $$\chi^{2}\left(H_{1},H_{2}\right)=\sum_{I}\frac{{\left(H_{1}\left(I\right)-H_{2}\left(I\right)\;\right)}^{2}}{H_{1}\left(I\right)}$$
iii. The histogram intersection calculates the similarity of two discretized probability distributions (histograms), with possible values of the intersection lying between 0 (no overlap) and 1 (identical distributions).^35^
  (3) $$I\left(H_{1},H_{2}\right)=\sum_{I}\min\left(H_{1}\left(I\right),H_{2}\left(I\right)\;\right)$$
iv. The Hellinger distance is related to the Bhattacharyya coefficient *BC*(*H*_1_,*H*_2_) and is used to quantify the similarity between two probability distributions.^36^ The maximum Hellinger distance is 1, and in the case of best match with a Bhattacharyya coefficient of 1, the Hellinger distance is 0.
  (4) $$BC\left(H_{1},H_{2}\right)=\;\sum_{I}\sqrt{H_{1}\left(I\right)\;H_{2}\left(I\right)}\;$$
  (5) $$H\left(H_{1},H_{2}\right)=\sqrt{1-BC\left(H_{1},H_{2}\right)}$$

#### Pairwise Comparisons Between Thickness Maps: Cross-Correlation and Mean Absolute Error

We carry out this validation based on healthy controls. We have 30 OCT stacks from healthy controls that were used to synthesize 100 synthetic stacks (the “training set”) and a further 100 OCT stacks from healthy controls that were not used in synthesizing the data (the “validation set”). To assess the degree of variation between thickness maps in healthy controls, we computed the maximum value of the cross-correlation between pairs of thickness maps, one taken from the validation set and one from the training set. This gave us a “real” distribution of maximum cross-correlations that we could then compare with synthetic data (i.e., the distribution of values when one thickness map is taken from the validation set and the other from the synthetic set). We also did a similar analysis using the mean absolute error (difference in the thickness of a given layer between a pair of maps). Again, the training and validation real maps were used to assess the distribution of mean absolute error in real maps, and this was then compared with the distribution obtained when synthetic maps were used instead of the real maps. We also carried out this analysis comparing the synthetic maps with the training set they were generated from, in order to see whether the synthetic maps matched these more closely than the cross-validation set.

#### Synthetic OCT Image Generation as a Data Augmentation Strategy for Diagnostic Tasks

The last validation strategy is based on a standard classification to validate the ability of the synthesis model as a data augmentation method. We train classification models using training sets containing only real maps versus using training sets augmented with our synthetic data in order to determine if the latter leads to better predictive performance to distinguish either MS or NMO from HC. Of the available eight retinal thickness maps, the first and second maps (from mRNFL and GCIPL) and the total macular thickness are the layers that discriminate best between HC, MS, and NMO, according to the literature.^9^^–^^16^ Each of these three thickness maps has a size of 512 × 25 pixels, and we used PCA to reduce the dimension of each map to 5. Overall, we construct a 15*D* space as input features for classification models. We train two types of binary classifiers, one to discriminate HC from MS and one for classifying HC from NMO, using a support vector machine (SVM) with radial basis kernel functions in both cases. A stratified fivefold cross-validation was used to evaluate the predictive performance of these models with a nested cross-validation for hyperparameter tuning (C and gamma) based on grid search. The partition into folds was done using the real data only, and in cross-validation iterations when a fold was used for training, we enhanced it with our synthetic data for the experiments with augmentation. This ensured that all test predictions were done on the real data and that the experiments with or without augmentation used the same partitions.

## Results

The results from the proposed three validation strategies are presented in this section. Overall, these aim to determine whether the synthesized OCT boundaries are proper representatives of the real ones and whether they can be used as an augmentation method in future research.

### Results of Histogram-Based Validation

Four metrics, including correlation coefficient, chi-square distance, histogram intersection, and Hellinger distance, are used to quantify the similarity of pairs of normalized histograms in the real training set (*H*_1_) and synthetic data (*H*_2_).

**Figure 7.**
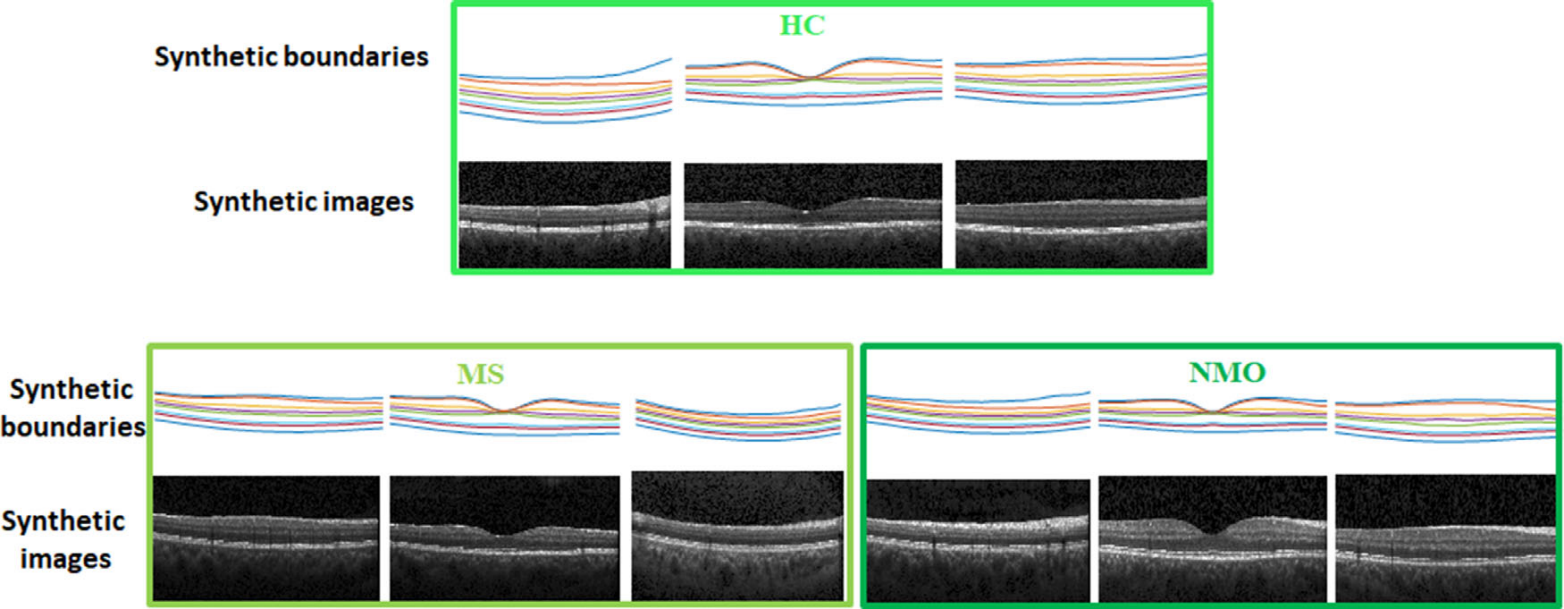
Three examples of synthetic images and corresponding boundaries from the three classes.

Figure 7 shows three samples of the generated data with corresponding boundaries in three classes. It should be emphasized that the boundaries are the only necessary data in this article due to the focus on retinal thickness maps rather than B-scans. Therefore, the synthetic images in Figure 7 are provided only for illustrative purposes and are not used in validation stages.

Five real OCT volumes are randomly selected from each class (HC, MS, and NMO) and 25 three-dimensional retinal layer boundary images are synthesized. From each 3D boundary image, thickness maps of seven retinal layers (mRNFL, GCIPL, INL, OPL, ONL MEZ, RPE) and the thickness of the total macula are calculated (each with a size of 512 × 25 pixels). The five thickness maps of real data with 5 × 512 × 25 values and 25 thickness maps of synthetic data with 25 × 512 × 25 values are then fed into a two-sample *t*-test. The *P* values reported in Table 1 show that real and synthetic data are not significantly different for any layer.

**Table 1.** Comparison of Thickness of Different Macular Layers Between Groups (Mean ± SD)

Characteristic	Total Macula	mRNFL	GCIPL	INL	OPL	ONL	MEZ	RPE
Real HC data	310.9 ± 12.8	29.7 ± 3.1	76.6 ± 2.8	35.7 ± 1.9	27.8 ± 2.1	61.5 ± 5.0	23.8 ± 0.5	55.5 ± 3.2
Synthetic HC data	308.0 ± 5.0	29.8 ± 1.9	75.8 ± 2.2	35.4 ± 1.7	28.2 ± 1.5	59.8 ± 3.5	23.8 ± 0.3	54.9 ± 1.2
*P* value	0.738	0.094	0.691	0.319	0.185	0.458	0.824	0.115
Real MS data	276.1 ± 11.3	24.3 ± 7.2	55.2 ± 6.6	33.8 ± 2.5	26.7 ± 0.9	58.6 ± 2.4	23.5 ± 0.3	53.8 ± 1.1
Synthetic MS data	275.3 ± 9.9	23.8 ± 7.8	55.2 ± 3.9	33.9 ± 2.7	26.6 ± 0.5	58.3 ± 1.9	23.5 ± 0.2	53.7 ± 0.9
*P* value	0.071	0.530	0.196	0.712	0.851	0.364	0.097	0.591
Real NMO data	281.2 ± 14.9	26.1 ± 6.8	56.2 ± 8.9	34.3 ± 4.3	29.6 ± 1.8	57.1 ± 2.4	23.6 ± 0.4	54.1 ± 3.0
Synthetic NMO data	282.5 ± 16.2	25.6 ± 7.6	8.76 ± 8.0	34.0 ± 2.3	27.3 ± 1.3	59.3 ± 2.9	23.6 ± 0.2	53.9 ± 1.1
*P* value	0.831	0.246	0.563	0.439	0.782	0.347	0.081	0.511

The normalized histograms are also compared between different pairs of real training data in Table 2. Table 3 reports the comparison between real and synthetic data from the same class. Finally, Table 4 reports the comparison between real and synthetic data from different classes.

**Table 2.** Averaged Metrics between All Possible Pairs of Normalized Histograms in the Real Training Set (Values from First and Second Maps [mRNFL and GCIPL] and the Total Macular Thickness)

	Total Macula			mRNFL			GCIPL		
Characteristic	HC	MS	NMO	HC	MS	NMO	HC	MS	NMO
Correlation coefficient	0.988	0.977	0.979	0.971	0.988	0.989	0.979	0.997	0.988
Chi-square distance	0.145	0.127	0.097	0.224	0.171	0.138	0.124	0.096	0.175
Histogram intersection	0.973	0.984	0.993	0.994	0.984	0.997	0.994	0.986	0.992
Hellinger distance	0.153	0.182	0.132	0.121	0.137	0.181	0.177	0.122	0.201

**Table 3.** Averaged Metrics Between All Possible Pairs of Normalized Histograms in the Two Groups of the Real Training Set and Synthetic Data from the Same Class (Values from First and Second Maps [mRNFL and GCIPL] and the Total Macular Thickness)

	Total Macula			mRNFL			GCIPL		
Characteristic	HC	MS	NMO	HC	MS	NMO	HC	MS	NMO
Correlation coefficient	0.982	0.972	0.968	0.955	0.970	0.969	0.971	0.986	0.981
Chi-square distance	0.159	0.173	0.136	0.219	0.193	0.122	0.146	0.142	0.129
Histogram intersection	0.969	0.989	0.984	0.981	0.987	0.966	0.962	0.985	0.975
Hellinger distance	0.161	0.172	0.164	0.211	0.205	0.139	0.192	0.126	0.182

**Table 4.** Averaged Metrics between Pairs of Normalized Histograms in the Two Groups of the Real Training Set and Synthetic Data from Different Classes (Values From First and Second Maps [mRNFL and GCIPL] and the Total Macular Thickness)

	Total Macula			mRNFL			GCIPL		
Characteristic	HC (Real) and MS (Synthetic)	HC (Real) and NMO (Synthetic)	MS (Real) and NMO (Synthetic)	HC (Real) and MS (Synthetic)	HC (Real) and NMO (Synthetic)	MS (Real) and NMO (Synthetic)	HC (Real) and MS (Synthetic)	HC (Real) and NMO (Synthetic)	MS (Real) and NMO (Synthetic)
Correlation coefficient	0.797	0.837	0.957	0.968	0.959	0.975	0.739	0.793	0.976
Chi-square distance	0.409	0.357	0.147	0.214	0.236	0.199	0.419	0.430	0.168
Histogram intersection	0.763	0.737	0.933	0.910	0.930	0.969	0.721	0.665	0.974
Hellinger distance	0.307	0.325	0.182	0.263	0.225	0.176	0.461	0.368	0.195

### Results of Pairwise Comparisons Between Thickness Maps

**Figure 8.**
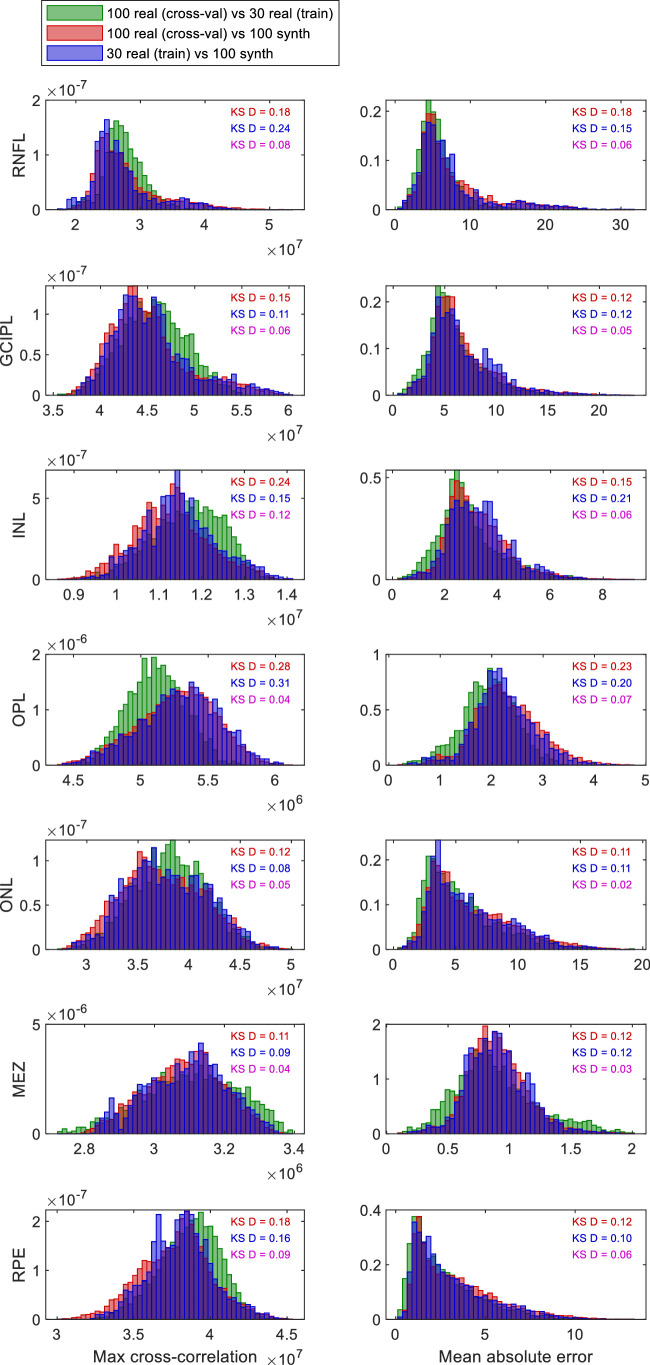
Comparison of pairwise cross-correlations (*left column*) and mean absolute errors (*right column*) for real versus synthetic thickness maps of the different retinal layers (*rows*). The *green* histograms show the distribution of the specified metric comparing pairs of real thickness maps: one from the cross-validation set of 100 maps and one from the training set of 30 maps. All pairs were used, so the histogram is based on 3000 pairwise comparisons. The *red* histograms show the distribution for pairs where one is again from the cross-validation set and one is from the set of 100 synthetic maps generated from the training set (10,000 comparisons). Ideally, the *red* and *green* distributions would be identical, but they are not. The *blue* histograms show the distribution for pairs where one is from the training set and one is synthetic (3000 comparisons). The text in *red* shows the Kolmogorov–Smirnov “D” statistic between the *red* and *green*, *blue* and *green*, and *blue* and *red* distributions (all are very highly significantly different from zero given the large number of pairwise comparisons).

Figure 8 shows the distribution of the peak cross-correlation (left columns) and the mean squared error (right columns) when comparing pairs of thickness maps for different layers. The green histograms give the distribution expected for real maps, as a reference. The red histograms compare the distributions encountered when comparing synthetic maps with real maps (from the cross-validation set; i.e., real maps that were not used in the generation of the synthetic maps). Ideally, these distributions would be identical, with a Kolmogorov–Smirnov (KS) “D” statistic of 0, but in fact they differ (KS-D shown on each panel; all are highly significant). However, the deviations are generally fairly small, and the modes are generally similar.

The blue histograms show the distributions when synthetic maps are compared with the training set of real maps they were generated from. One might have expected that these would agree more closely, thus producing higher cross-correlation and lower mean absolute error. However, no such effect is apparent, and the KS-D between this and the real/real green distributions is in fact slightly smaller than when the cross-validation set is used. The KS-D between the red and blue distributions is shown last, in pink. Unsurprisingly, these values are much lower, since we are now comparing two real/synth distributions, although in fact still significant.

Overall, this analysis has revealed significant differences between the thickness map from real versus synthetic boundaries. However, the agreement seems acceptable. The distribution of the pairwise comparison metrics is very similar between pairs of real maps as between real/synthetic pairs.

### Results of Classification-Based Validation

A pilot evaluation is done using two binary SVM classifiers with radial basis kernel functions (one to discriminate HC from MS and one to discriminate HC from NMO) with the hyperparameters tuned using grid search. From each of the three classes (HC, MS, and NMO), thirty 3D OCT volumes were randomly selected, and the dimension of each OCT volume is reduced to 15 using the PCA algorithm (as elaborated above). The classification algorithms are trained twice, once with real data and once with the synthesized data. Stratified fivefold nested cross-validation is used for splitting training/test partitions.

For classification of MS from HC, in the first trial, the real data indices for fivefold cross-validation were used for training. The metrics are reported in Table 5 (real training data). In the second trial, in each splitting iteration, 24 OCT scans for each class (whose indices are determined by fivefold algorithm as the training data) were selected. The 24 HC OCT scans were used by one synthesis model, and the 24 MS OCT scans were used by another synthesis model, each producing new maps similar to their own inputs. Different numbers of synthesized maps were produced using synthesis models for each category and added to the sets of 24 real maps. The metrics from the second trial are reported in Table 5.

**Table 5.** Comparisons of Classification Results for MS/HC Discrimination Between the Real Training Data and Real Data Plus Synthetic OCTs with the Proposed Method, for Different Numbers of Synthetic OCTs

Characteristic	Sensitivity	Specificity	Precision	False-Positive Rate	False-Negative Rate	Accuracy	F1 Score
Real training data (24 OCT scans for each class)	0.7333	0.6154	0.6875	0.3846	0.2667	0.6786	0.7097
Real plus 24 × 1 = 24 synthetic data	0.7667	0.6638	0.7188	0.3462	0.2813	0.7143	0.7419
Real plus 24 × 2 = 48 synthetic data	0.8000	0.6923	0.7500	0.3077	0.2000	0.7500	0.7742
Real plus 24 × 4 = 96 synthetic data	0.8333	0.7692	0.8065	0.2307	0.1667	0.8036	0.8197
Real plus 24 × 8 = 192 synthetic data	0.8421	0.7586	0.8205	0.2414	0.1579	0.8060	0.8312
Real plus 24 × 16 = 384 synthetic data	0.8333	0.7742	0.8333	0.2258	0.1667	0.8082	0.8333

**Figure 9.**
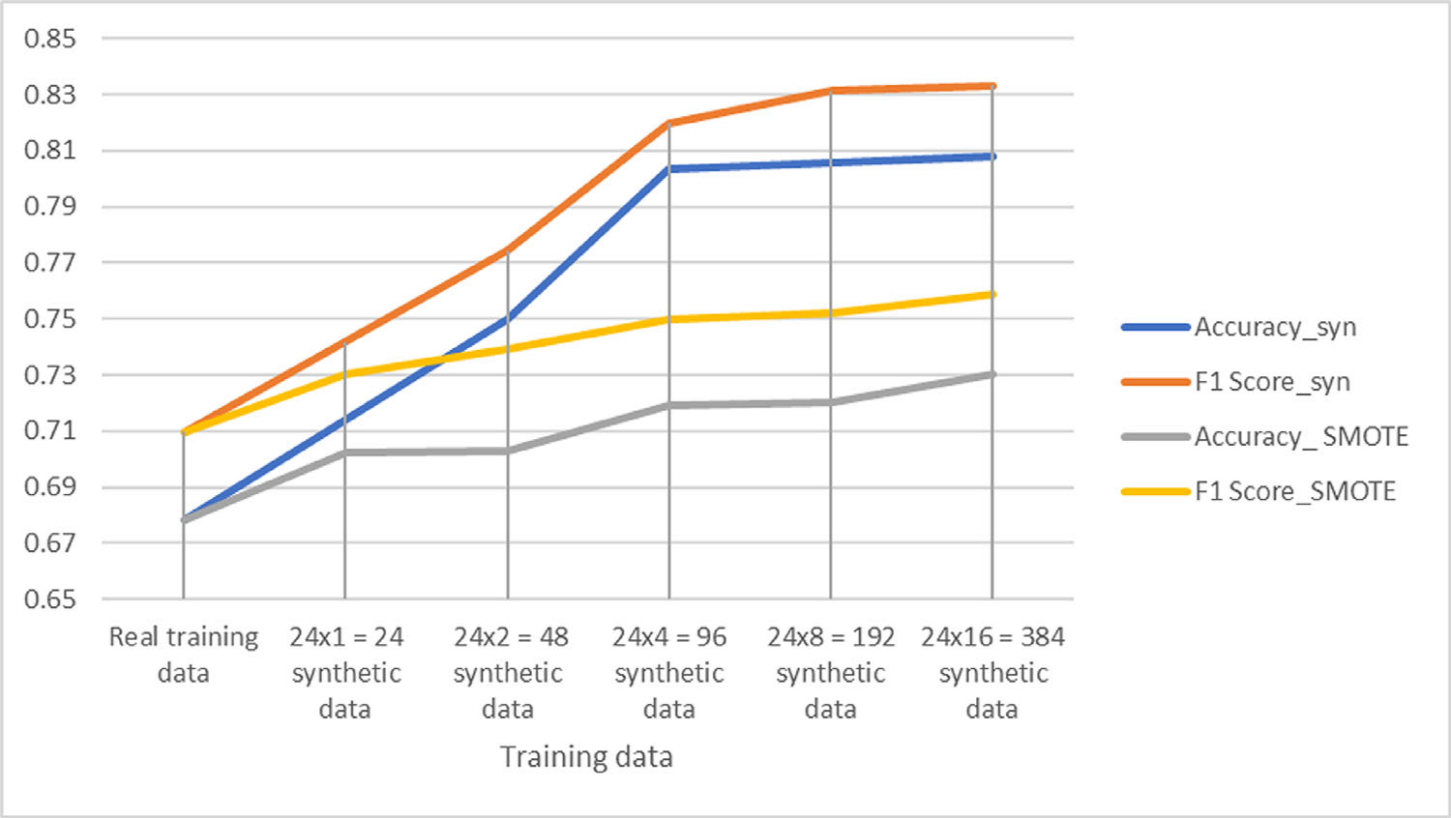
Classification performance for MS/HC discrimination for different training sets: real training data, real data with different numbers of additional synthetic OCTs, and real data with different numbers of data added via SMOTE.

Furthermore, to compare the performance of the method with oversampling methods such as SMOTE,^37^ instead of synthesizing full 3D OCT data and then generating thickness maps to extract the final feature vector for the classification model, we directly resampled from the thickness map of the annotated training set to generate additional sample points for training in Figure 9. For this purpose, we use SMOTE to produce the synthetic points, generating a number of synthetic points equal to that of our method in order to have a fair comparison. Accuracy and F1 score are compared, and the results indicate that using SMOTE in this way also effectively enhances the training set, and the samples generated by our method lead to better performance in all scenarios. The rationale behind this result is that the oversampling technique assumes that the feature space behaves as a Euclidean space with equal relevance for each axis, so that distances are meaningful.^38^ The thickness maps may not fully fulfill this assumption.

**Figure 10.**
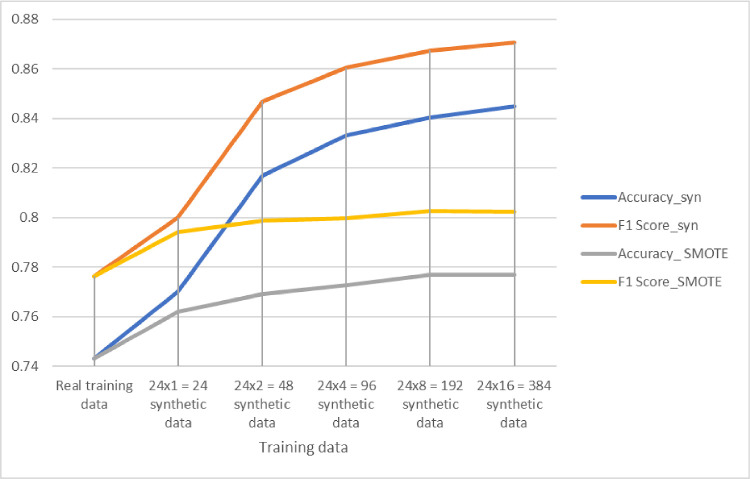
Classification performance for NMO/HC discrimination for different training sets: real training data, real data with different numbers of additional synthetic OCTs, and real data with different numbers of data added via SMOTE.

Similarly, the same procedure was repeated for classification of HC and NMO data, and the results are shown in Table 6 and Figure 10.

**Table 6.** Comparisons of Classification Results for NMO/HC Discrimination Between the Real Training Data and Real Data Plus Synthetic OCTs with the Proposed Method, for Different Numbers of Synthetic OCTs

Characteristic	Sensitivity	Specificity	Precision	False-Positive Rate	False-Negative Rate	Accuracy	F1 Score
Real training data (24 OCT scans for each class)	0.7857	0.6875	0.7674	0.3125	0.2143	0.7432	0.7765
Real plus 24 × 1 = 24 synthetic data	0.8095	0.7188	0.7907	0.2813	0.1905	0.7703	0.8000
Real plus 24 × 2 = 48 synthetic data	0.8571	0.7586	0.8372	0.2414	0.1429	0.8169	0.8471
Real plus 24 × 4 = 96 synthetic data	0.8810	0.7667	0.8409	0.2333	0.1190	0.8333	0.8605
Real plus 24 × 8 = 192 synthetic data	0.8780	0.7857	0.8571	0.2143	0.1220	0.8406	0.8675
Real plus 24 × 16 = 384 synthetic data	0.8810	0.7931	0.8605	0.2069	0.1190	0.8451	0.8706

## Discussion and Conclusion

In this study, we have shown (1) that the proposed 3D ASM synthesis model can generate realistic-appearing synthetic maps of retinal layer boundaries, (2) that the histogram-based validation shows the histogram of generated 2D maps (retinal thickness maps) corresponds to the histogram of the maps in the training database, (3) that cross-correlations between the generated and real 2D maps shows the validity of the synthetic data, and (4) a standard classification-based validation confirms the efficacy of synthetic data as an augmentation method for training the classification algorithms.

The proposed model is general, and by changing the weights vector, it can generate new examples of different diagnostic classes. However, the realistic shape (resembling layer boundaries from real OCT maps) is controlled by imposing limitations on the weights vector. Figures 4 to 6 visually depict 2D maps of synthetic thickness maps and show their similarity to real data. The similarity between real and synthetic data in horizontal B-scans is also demonstrated in Figure 7. Despite the subtlety of differences between the three classes (HC, MS, NMO), which often elude expert clinicians, the numerical evaluations prove that the proposed model is able to capture them.

The normalized histograms are compared between different pairs of the data in Tables 2, 3, and 4. Higher correlation coefficients and histogram intersections, as well as lower chi-square distances and Hellinger distances, indicate greater similarity in the compared probability distributions. Comparison of Table 2 with Table 3 indicates that the similarity between all synthetic–real pairs of histograms in the same disease class is as great as the similarity between real–real histogram pairs. In Table 4, pairs of normalized histograms are drawn from the real and synthetic data, across disease classes. These values indicate that real MS and synthetic NMO data are similar in their normalized thickness histograms for total retina, mRNFL, and GCIPL. However, the difference between HC and disease data is captured by the synthetic data, in that the similarity is reduced between HC (real)/MS (synthetic) and HC (real)/NMO (synthetic) total macular volume and GCIPL thickness, in keeping with earlier clinical studies^9^^–^^16^ that indicate the greatest effect of disease on those parameters.

The cross-correlation analysis is more sensitive, because it assesses not only the distribution of thickness values in a layer but also the pattern of thickness across the retina. This method did reveal small but significant differences between the synthetic maps and real maps. More work will be needed to understand and correct these discrepancies.

However, even in its current state, we demonstrate that the proposed model is demonstrably useful as an augmentation method, since including synthetic examples improved performance. Our method also avoids the problem of mode collapse observed in other synthetic image construction algorithms like generative adversarial networks (GANs). Provided different weights are used, the ASM model is guaranteed to generate different synthetic data points. The intradiversity of the method was also demonstrated in our correlation-based validation: the distribution of cross-correlation values was similar when synthetic thickness maps were compared with (a) the real “training” maps used to generate them (blue histograms in Fig. 8) as with (b) a distinct set of real “cross-validation” maps (red histograms). If the synthetic maps remained very close to the real maps used to generate them, we would have expected systematically higher correlations in the former comparison. Furthermore, the complexity of the proposed method is lower than GAN methods, and it is able to work with a very small training data set, which is not practical for GANs.

Finally, one of the important outcomes of the proposed synthetic data is that we can provide large amounts of data for training ML algorithms without the privacy concerns that affect real human data, since retinal images are considered protected health information.^39^ The method could be expanded to produce synthetic OCT image data with different features ranging from age, ethnicity, and severity (e.g., for use in tele-education platforms).^40^ Thus, the ASM model has potential for aiding future education of neuro-ophthalmology trainees.

In conclusion, the proposed method for generating synthetic OCT data can address the problem of limited data sets from patients with neurodegenerative disease, which could help us apply ML approaches even when OCT data are limited. The degree of matching between corresponding layers in synthesized data reflects the realism of the synthetic data and justifies its use to augment real data in future research.

## Supplementary Material

Supplement 1
