# Supplementary material for: Synthetic OCT Data Generation to Enhance the Performance of Diagnostic Models for Neurodegenerative Diseases
Source: Transl Vis Sci Technol. 2022 Oct 6;11(10):10. doi: 10.1167/tvst.11.10.10 (PMC9554224; doi:10.1167/tvst.11.10.10)
Supplement: Supplement 1 [file tvst-11-10-10_s001.pdf]

Supplementary to

Synthetic OCT data generation to enhance the performance of diagnostic models for neurodegenerative diseases

This supplement elaborates the details of the proposed method to synthesize OCT boundaries.

We apply a Procrustes analysis [30] to align all image-stacks to a reference image-stack. This involves applying a 2D translation to equalize the mean *x* and *y* (the mean *z* is already constant for all stacks), a rotation about the *z* axis and a rotation about the *x* axis. We do not apply any scaling.

A Point Distribution Model (PDM) [31] is then constructed. The *i*th image-stack is now represented by an 54,600-element vector $\boldsymbol{X_i}$ describing the *x,y,z* locations of 91 landmark points on each of the 8 boundaries in each of the 25 Bscans (3 x 91 x 8 x 25 = 54,600). The set of *N* image-stacks now form a cloud in this 54,600-dimensional space. Assuming that the variability within the population occurs along just a few directions in this space, the dimensionality can be reduced to a lower space using Principal Component Analysis (PCA). For this purpose:

1. Define $\overline{\boldsymbol{X}}$ to be the mean value of all the image-stack vectors $\boldsymbol{X_i}$:

   $$\overline{\boldsymbol{X}} = \frac{1}{N}\sum_{i=1}^{N}\boldsymbol{X}_i \qquad (1)$$

2. Divide each image-stack vector by its standard deviation, and subtract off the mean so as to obtain centered, scaled data ($\boldsymbol{Z}_i$)
3. Calculate the covariance matrix of the centered, scaled data by:

   $$S = \frac{1}{N-1}\sum_{i=1}^{N}\boldsymbol{Z}_i\,\boldsymbol{Z}_{\boldsymbol{i}}^T \qquad (2)$$

4. Calculate the eigenvectors and eigenvalues of the covariance matrix S, ordered by descending eigenvalues. Because the number of observations, *N,* is less than the number of dimensions, the number of eigenvectors/values is (*N*-1). The variance explained by the *i*th eigenvector $\boldsymbol{p_i}$ is equal to the corresponding eigenvalue λ_i. We find that between 4 and 28 eigenvectors are required to account for at least 95% of the variance, depending on the number of image-stacks *N* and their variability. Empirically, we have found good results by using the first 7

eigenvectors. Therefore, after dimension reduction, the original 54,600-dimensional space has been approximated by a space of dimension 7.

Each layer boundary in the training set can now be approximated by the mean shape plus a weighted sum of the first t principal components:

$$\boldsymbol{X} \approx \overline{\boldsymbol{X}} + P\boldsymbol{b} \tag{3}$$

where $P = (\boldsymbol{p}_1, \boldsymbol{p}_2, \boldsymbol{p}_3, \ldots, \boldsymbol{p}_t)$ is the matrix formed by the first t eigenvectors, and $b = (b_1, b_2, b_3, \ldots, b_t)$ is the weights vector. Now, we can synthesize new layer boundaries by allocating different numbers to elements of b. However, arbitrary values of b could result in an implausible shape which would not arise from a real OCT scan. The real OCT maps occupy an approximately Gaussian point cloud with standard deviation $\sqrt{\lambda_i}$ along each axis. When generating synthetic data, we therefore draw b_i from a Gaussian distribution with mean 0 and standard deviation $\sqrt{\lambda_i}$. We also impose a hard limit of

$$-3\sqrt{\lambda_i} \leq b_i \leq +3\sqrt{\lambda_i} \tag{4}$$
